# Serological assessment of the establishment of herd immunity against measles in a health district in Malaysia

**DOI:** 10.1186/s12879-016-2069-y

**Published:** 2016-12-08

**Authors:** Y. Hazlina, M. A. Marlindawati, K. Shamsuddin

**Affiliations:** 1 Department of Community Health, Faculty of Medicine, Universiti Kebangsaan Malaysia Medical Centre (UKMMC), Jalan Yaacob Latiff, Bandar Tun Razak, Cheras, 56000 Kuala Lumpur, Malaysia; 2 Department of Pathology, Hospital Tuanku Jaafar, Jalan Rasah, 70300 Seremban, Negeri Sembilan Malaysia

**Keywords:** *Measles*, *Measles IgG antibody*, *Seroprevalence*, *Herd immunity*

## Abstract

**Background:**

Malaysia still faces challenges optimizing resources to effectively eliminate measles through high immunization and herd immunity, with sporadic outbreaks of measles as evidence. The objective of this study is to determine the age-specific positive measles antibodies seroprevalence used for assessing the establishment of herd immunity against measles in different age groups. This is useful for identifying vulnerable age groups requiring supplementary immunization.

**Methods:**

A seroprevalence study was conducted among respondents aged 6–9 years, 15–24 years and 45–54 years attending government health clinics in Seremban between September 2014 and January 2015. A total of 1541 measles IgG antibody status were determined using ELISA technique (NovaTec Immundiagnostica GMBH) and assessment of establishment of herd immunity was based on indicators developed by Plans. Data on socio-demographic background as well as medical and medication history were also gathered.

**Results:**

Seropositive rate for all respondents were 87% (95% CI 85–89), while the rest had either indeterminate [6% (95% CI 5–7)] or negative titre [7% (95% CI 6–8)]. None of the factors analyzed except for age were significant predictors of positive measles antibodies. Seropositive rate differed by age with the highest rate seen in adults (94%; CI 92–96), followed by children (90%; 95% CI 87–94) and adolescents, and young adults (74%; 95% CI 70–78). Based on Plans’ indicators, herd immunity was established in adults and children, but not in adolescents and young adults.

**Conclusions:**

To tackle the most susceptible group in the present study, it is advisable to give booster vaccination to secondary school students and freshmen who enter colleges and universities in Malaysia.

## Background

Measles is a highly contagious disease affecting more than 95% of exposed populations before the introduction of measles containing vaccine (MCV). In 1980, almost 2.6 million children aged below 6 years were estimated dead secondary to measles annually [1]. Despite all the progress in measles control, many countries are still facing difficulties managing resources to protect their children from this disease burden effectively. Therefore, measles continues to be a leading cause of vaccine preventable mortality among children [2].

Malaysia was reported to be the country in the Western Pacific Region with the highest confirmed measles incidence with a rate of 63.7 per 1,000 000 populations in 2012 [3]. In Seremban, the incidence was higher with a rate of 72.1 per 1,000 000 populations. Since 1982 until 2002, a single dose measles vaccination was given to children at 9 months of age. Subsequently in 2002, the Malaysian Ministry of Health implemented measles elimination strategy when a double dose measles-mumps-rubella (MMR) vaccine was introduced to children at 12 months and 7 years old [4].

Vaccination limits the number of secondary persons who can be infected. Hence, we need to reduce the number of unimmuned person so that outbreaks could not occur [5]. The ultimate aim of the use of vaccination in infectious diseases field is disease elimination where persistent transmission no longer exists in the population and secondary infection due to imported cases is terminated [6]. In order to assess immunization programmes for measles, it is beneficial if the level of antibody against measles is determined. Elimination of indigenous measles could be obtained if the prevalence of susceptible individuals remains at low level [7].

### Study objectives

The main objectives of this study were to evaluate the establishment of herd immunity against measles among subgroups of population by comparing their seroprevalence of positive measles antibodies (*p*) with critical prevalence of antibody associated with herd immunity (p_c_) according to Plans [8].

## Methods

### Sampling population

This was an institutional ethics approved cross-sectional study conducted among children, adolescents and young adults, and adults attending government/ public health clinics in Seremban, the capital of the state of Negeri Sembilan from September 2014 to January 2015. Seremban was chosen as s study site since the incidence rate was high. It is also the biggest district in Negeri Sembilan with the highest population having both urban and rural health clinics. The sample size calculation for this study was based on comparison of two proportions [9] with power set at 80%, two-sided confidence level at 95% and using the comparative prevalence of a local study conducted by Saraswathy et al. [10]. The sampling of patients was done using multistage sampling. In the first stage, 10 of the 11 health clinics in the district of Seremban were selected using non-probability purposive sampling technique. In the second stage, samples were stratified into three age groups; children (6–9 years old), adolescents and young adults (15–24 years old), and adults (45–54 years old) similar to the age group of the study conducted by Plans [8]. Every 5th patient attending the selected health clinics were recruited into the study according to the stratified age.

About 5 mls of blood sample were collected in a plain tube, followed by extraction of 1 mls of serum which was kept deep frozen between -20° and -70 °C until analysed. Other factors like socio-demographic background as well as medical and medication history were collected through face to face interview with respondents/ parents or guardians using a standardized and validated questionnaire. The measles IgG antibody status were determined using ELISA (Enzyme-linked Immunosorbent Assay) technique (NovaTec Immunodiagnostica GMBH) [11] at the accredited Pathology Laboratory, Hospital Tuanku Jaafar. According to the manufacturer’s information, the sensitivity and the specificity of the measles IgG kit were more than 95% [11].

### Data analysis

The serological findings were classified as seronegative when measles IgG level was less than 120 IU/ml, indeterminate when the measles IgG level was between 120 and 220 IU/ml and seropositive when measles IgG level was more than 220 IU/ml. For statistical analysis, the respondents were classified into those who were seropositive and those who were not (indeterminate and seronegative).

Descriptive and analytical analyses of data were carried out using ‘Statistical Package for Social Sciences’ (SPSS) Version 20.0. Statistical significance level was taken at the *p* value < 0.05 and 95% confidence intervals (CI). Descriptive statistics such as frequency and percentage were used to analyse the socio-demographic variables. The binary logistic regression analysis was used to determine the association between the variable of interest and positive measles antibody in the respondents. If significant association between variables were found, the Odds Ratio value will be used to determine the association between the variable in question and positive measles antibody.

Finally, the establishment of herd immunity was determined based on Plans’ indicator [8]. Herd immunity was established when the prevalence of antibodies observed in a serological study (*p*) was higher than the critical prevalence of antibodies associated with herd immunity (p_c_); *p* > p_c.._ Herd immunity was not established when the prevalence of antibodies observed in a serological study (*p*) was lower than the critical prevalence of antibodies associated with herd immunity (p_c_); *p* < p_c_, where *p* was the positive seroprevalence of measles IgG and p_c_, the critical prevalence of antibody against measles was determined using a mathematical model developed by Plans [8]. In Plans’ formula, *p* was also used to estimate the critical prevalence of antibody against measles (p_c_) in the population and by subgroups (age, gender and ethnicity) as shown in the formulae.

### Seroprevalence of positive measles antibody (p)

The variable (*p*) denoted the presence of antibody against measles (positive measles IgG) in the study population. The prevalence (*p*), is also used to calculate PPV, a component for the estimation of critical prevalence of antibodies associated with herd immunity (p_c_) as shown in Formula 1.

### Critical prevalence of antibodies associated with herd immunity (p_c_)

The estimation of critical prevalence of antibodies associated with herd immunity (p_c_) was based on Formula 1. The critical prevalence of antibodies associated with herd immunity (p_c_) could be determined from the prevalence of protected individuals or herd immunity threshold (I_c_), the sensitivity (Se) and predictive value of positive serological result (PPV) [8]:

$$ {\mathrm{p}}_{\mathrm{c}} = {\mathrm{I}}_{\mathrm{c}}\mathrm{S}\mathrm{e}\ /\ \mathrm{P}\mathrm{P}\mathrm{V}\left(\mathrm{Formula}\ 1\right) $$

where;

- Ic = Herd immunity threshold equals 92.5% based on Plans’ indicator [8]
- Se = Sensitivity of the serological test equals more than 95% based on manufacturer’s data
- PPV = Predictive value of positive serological result

### Herd immunity threshold (I_c_)

Herd immunity threshold (I_c_) was defined as the minimum prevalence of individuals who are protected necessary to cease the transmission of an infection in the population [12, 13].

$$ {\mathrm{I}}_{\mathrm{c}} = {\mathrm{R}}_{\mathrm{o}}{\textstyle \hbox{-} }\ 1/\ {\mathrm{R}}_{\mathrm{o}}=1\ {\textstyle \hbox{-} }\ 1/\ {\mathrm{R}}_{\mathrm{o}}\ \left(\mathrm{Formula}\ 2\right) $$

I_c_ is 91-94%, of which the mean was 92.5% based on Plans [8] with R_o_, basic reproductive number for measles of 11 – 18.

### Sensitivity of the serological test (Se)

This was more than 95% according to manufacturer’s data [10].

### Predictive value of positive serologic result (PPV)

In this formula, the predictive value of positive serologic result could be obtained from the prevalence of antibodies (*p*), the sensitivity (Se) and the specificity (Sp) of the serologic test:

$$ \mathrm{P}\mathrm{P}\mathrm{V} = \left(p\mathrm{S}\mathrm{e}\right)/\left[\left(p\mathrm{S}\mathrm{e}\right) + \left(1-p\right)\ \left(1-\mathrm{S}\mathrm{p}\right)\right]\ \left(\mathrm{Formula}\ 3\right) $$

## Results and discussion

## Results

### Socio-demographic characteristics and medical/ medication history of participants

A total of 1541 of the 1712 (90%) patients approached agreed to participate in this study. Table 1 shows that most of the respondents were adults aged 45–54 years (48.9%), followed by adolescents and young adults aged 15–24 years (31.7%) and children aged 6–9 years (19.3%). The numbers of female participants were slightly higher (53.2%) than male participants (46.8%). A total of 927 (60.2%) participants were Malays, 74.2% had lower education and 75.1% had lower household income. Lower educational level was defined as those who did not go to school or had received at most secondary education whereas lower household income was defined as average total income for all household members in Malaysian Ringgit (MYR) of less than RM 4000 for a typical month. The respondents were mostly born in places outside Seremban (54.4%) and in urban locality (66%). Of all respondents, 81.6% attended urban located health clinics in Seremban district and most of them had no medical problems (61.1%) and were not on any medications (63.3%).

**Table 1:** Association between socio-demographic characteristics and medical/ medication history and seroprevalence of measles antibodies (measles IgG titre), (*n* = 1541)

Characteristics	Seroprevalence of measles IgG titre							
	N(%)	Positive titre^a^ (95% CI)		Indeterminate and negative titres^b^ (95% CI)		Crude odds ratio (95% CI)		*p* value
Total	1541(100)	87%	(85–89)	13%	(11–15)			
Age (year)								
[6–9]	298 (19.3)	90%	(87–94)	10%	(6–13)	1.00		
15–24	489 (31.7)	74%	(70–78)	26%	(22–30)	0.31	(0.20 – 0.47)	0.0001
45–54	754 (48.9)	94%	(92–96)	6%	(4–8)	1.74	(1.07 – 2.84)	0.027
Gender								
[Male]	721 (46.8)	85%	(83–88)	15%	(12–17)	1.00		
Female	820 (53.2)	88%	(86–91)	12%	(9–14)	1.30	(0.97 – 1.75)	0.083
Ethnicity								
[Non-Malay]	614 (39.8)	89%	(87–92)	11%	(8–13)	1.00		
Malay	927 (60.2)	86%	(83–88)	14%	(12–17)	0.73	(0.54–1.00)	0.050
Education Level								
[Lower]	1144 (74.2)	88%	(87–90)	12%	(10–13)	1.00		
Higher	397 (25.8)	83%	(79–87)	17%	(13–21)	0.65	(0.47–0.89)	0.008
Household income								
[Lower]	1157 (75.1)	86%	(84–88)	14%	(12–16)	1.00		
Higher	384 (24.9)	89%	(86–92)	11%	(8–14)	1.29	(0.90–1.85)	0.170
Place of birth								
[Others]	839 (54.4)	89%	(87–91)	11%	(9–13)	1.00		
Seremban	702 (45.6)	85%	(82–88)	15%	(12–18)	0.71	(0.53–0.96)	0.024
Locality of place of birth								
[Urban]	1017 (66.0)	86%	(84–88)	14%	(12–16)	1.00		
Rural	524 (34.0)	89%	(86–92)	11%	(8–14)	1.30	(0.94–1.81)	0.110
Locality of health clinics attended								
[Urban]	1258 (81.6)	87%	(86–89)	13%	(11–14)	1.00		
Rural	283 (18.4)	86%	(81–90)	14%	(10–19)	0.85	(0.59–1.24)	0.404
Medical problems								
[No]	941 (61.1)	84%	(81–86)	16%	(14–19)	1.00		
Yes	600 (38.9)	92%	(90–94)	8%	(6–10)	2.22	(1.57–3.12)	0.0001
Medication history								
[No]	976 (63.3)	84%	(81–86)	16%	(14–19)	1.00		
Yes	565 (36.7)	93%	(90–95)	7%	(5–10)	2.41	(1.68–3.43)	0.0001

[] Reference group

^a^Positive titre: > 220 IU/ml

^b^Indeterminate titre 120–220 IU/ml and negative titre < 120 IU/ml

### Seroprevalence and associated factors of measles antibodies (measles IgG)

Table 1 also shows the percentages of respondents who were measles IgG seropositive, indeterminate or seronegative by different socio-demographic variables and medical/ medication history. The overall prevalence of positive titre for measles IgG in the sample surveyed was 87% (95% CI 85-89) while 6% (95% CI 5-7) of all respondents had indeterminate titre and another 7% (95% CI 6-8) had negative titre. The proportion of seropositive respondents differed by age and was highest in adults aged 45–54 years (94%; 95% CI 92-96) and lowest in teenagers and young adults aged 15–24 years (74%; 95% CI 70-78). The proportion of respondents with indeterminate and negative titre level was highest in teenagers and young adults aged 15–24 years (11%; 95% CI 9-14 and 15%; 95% CI 11-18) and lowest in adults aged 45–54 years (3%; 95% CI 2-4).

Differences in measles seropositive rate were seen by gender and ethnicity. Female respondents had higher positive titre (88%; 95% CI 86-91) in comparison to male respondents (85%; 95% CI 83-88). Measles seropositive rate was lowest in Malays (86%; 95% CI 83-88) and was highest in others (93%; 95% CI 83-103). Both Chinese and Indian had measles seropositive rate of 90% (95% CI 85-94) and 89% (95% CI 85-92) respectively. Differences were also seen by education level with respondents with lower education level having higher positive titre (88%; 95% CI 87-90), while those with higher education level had lower positive titre (83%; 95% CI 79-87).

Positive seroprevalence was also higher in respondents with higher household income (89%; 95% CI 86-92), born outside Seremban (89%; 95% CI 87-91) and rural born (89%; 86-92). Measles seropositive rate was slightly lower in respondents who attended rural health clinics (86%; 95% CI 81-91) in comparison to respondents who attended urban health clinics (87%; 95% CI 86-89). Obvious differences were also found in respondents with medical problems and medication history where both had higher positive titre for measles IgG (92%; 95% CI 90-94 and 93%; 95% CI 90-95).

The possible association between socio-demographic variables and medical/ medication history and prevalence of measles antibodies was analysed by initially conducting chi square test and further verified with simple logistic regressions. Simple logistic regression tests were conducted to screen for important independent variables prior to multivariable analysis. To test for the association of these socio-demographic and medical/ medication history, measles seropositive titre were compared.

Table 1 shows that there were significant differences in measles positive seroprevalence by age, education level, place of birth, medical problems and medication history (*p* < 0.05). Adolescents and young adults aged 15–24 years had decreased Odds of having positive measles IgG by 69% compared to children aged 6–9 years (OR 0.31; 95% CI 0.20-0.47) while adults aged 45–54 years had Odds ratio of 1.74 (95% CI 1.07-2.84) of having positive measles IgG in comparison to children aged 6–9 years.

Respondents with higher education level had decreased Odds of having positive measles IgG titre by 35% in comparison to those with lower education level (OR 0.65; 95% CI 0.47-0.89). Those born in Seremban had decreased Odds to be seropositive by 29% than those who were born outside (OR 0.71; 95% CI 0.53-0.96). Those with medical problems had Odds ratio of measles seropositive of 2.22 (95% CI 1.57-3.12) in comparison to those who gave no history of medical problems. Respondents with history of taking medications were also more likely to be seropositive than those who were not (OR 2.41; 95% CI 1.68-3.43).

For multivariable or adjusted analysis, after adjusting for significant variables explored in the crude analysis, only age group remained the strongest and significant predictor for seropositivity of measles IgG based on both forward selection and backward elimination strategies.

### Establishment of herd immunity against measles among all respondents

Herd immunity is established when *p* > p_c_. In the subsequent analysis, Table 2 shows three scenarios depending on how we dealt with the 6% indeterminate titre outcome of the 1541 respondents in the study.

**Table 2:** Seroprevalence of positive measles antibodies (*p*), critical prevalence of antibodies associated with herd immunity (p_c_) and establishment of herd immunity (HI) - comparing worst, best and absolute case scenario

Characteristics	Establishment of herd immunity (HI)								
	Worst Case^a^ (*n* = 1541)			Best Case^b^ (*n* = 1541)			Absolute Case^c^ (*n* = 1447)		
	*p* (%) (95% CI)	p_c_ (%) (range)	HI	*p* (%) (95% CI)	p_c_ (%) (range)	HI	*p* (%) (95% CI)	p_c_ (%) (range)	HI
Total	87.0 (85.0–89.0)	89.4 (87.9–90.8)	-	93.0 (92.0–94.0)	89.1 (87.6–0.5)	+	93.0 (91.0–94.0)	89.1 (87.6–90.5)	+
Age (year)									
6–9	90.0 (87.0–94.0)	89.2 (87.4–90.7)	+	95.0 (92.0–97.0)	89.0 (87.6–90.5)	+	95.0 (92.0–97.0)	89.0 (87.6–90.5)	+
15 – 24	74.0 (70–78)	90.4 (88.9–91.8)	-	85.0 (82.0–89.0)	89.5 (88.0–90.9)	-	84.0 (80.0–87.0)	89.5 (87.6–90.5)	-
45–54	94.0 (92.0–96.0)	89.0 (87.6–90.5)	+	97.0 (96.0 – 98.0)	88.9 (87.5–90.4)	+	97.0 (96.0–98.0)	88.9 (87.5–90.4)	+
Gender									
Male	85.0 (83.0–88.0)	89.5 (88.0–90.9)	-	92.0 (90.0–94.0)	89.1 (87.7–90.6)	+	92.0 (89.0–94.0)	89.1 (87.7–90.6)	+
Female	88.0 (86.0–91.0)	89.3 (87.9–90.7)	-	94.0 (92.0–96.0)	89.0 (87.6–90.5)	+	94.0 (92.0–95.0)	89.0 (87.6–90.5)	+
Ethnicity									
Malay	86.0 (83.0–88.0)	89.4 (87.9–90.8)	-	92.0 (91.0–94.0)	89.1 (87.7–90.6)	+	92.0 (90.0–94.0)	89.1 (87.7–90.6)	+
Chinese	90.0 (85.0–94.0)	89.2 (87.8–90.7)	+	93.0 (90.0–97.0)	89.1 (87.6–90.5)	+	93.0 (90.0–97.0)	89.1 (87.6–90.5)	+
Indian	89.0 (85.0–92.0)	89.3 (87.8–90.7)	-	94.0 (92.0–97.0)	89.0 (87.6–90.5)	+	94.0 (92.0–97.0)	89.0 (87.6–90.5)	+
Others	93.0 (83.0–103.0)	89.1 (87.6–90.5)	+	96.0 (89.0–104.0)	88.8 (87.4–90.4)	+	96.0 (89.0–104.0)	88.8 (87.4–90.4)	+

^a^Assuming all indeterminate results are negative,^b^ Assuming all indeterminate results are positive, ^c^Ineterminate result are not presented

(*p*) Seroprevalence of positive measles antibodies; (p_c_) Critical prevalence of antibodies associated with herd immunity

(*HI*) Herd Immunity; + Herd Immunity Established (*p* > mean p_c_); - Herd Immunity Not Established (*p* < mean p_c_)

Calcualted using Critical Prevalence of Protected Individuals, I_c_ (%) = 92.5 (91–94)

In the first case, considered as the worst case scenario, analysis of 1541 respondents and assuming all 94 indeterminate cases as seronegative gave us a *p*, seropositive rate of 87% and the calculated p_c_ based on Plans’ indicator was 89.4%. Thus *p* < p_c_ and herd immunity for the 1541 respondents was not established. Subsequent assessment of subgroups, the 15–24 years old respondents, both genders, in Malays and Indians also revealed that herd immunity were not established since *p* < p_c_. However, herd immunity was established among the 6–9 years old, 45–54 years old, and among Chinese and others since *p* > p_c_.

In the second scenario, considered the best case, analysing all 1541 respondents and assuming all 94 indeterminate measles IgG cases as positive, gave us a measles seropositive rate, *p* of 93%, while the calculated p_c_ based on Plans’ indicator was 89.1%. Hence, *p* > p_c_ and herd immunity for the 1541 respondents was established in the best case scenario. Subsequent assessment showed that the 6–9 and 45–54 years old, both genders and all ethnicities showed establishment of herd immunity since *p* > p_c_. Nevertheless, among the 15–24 years old, herd immunity was not established since *p* < p_c_.

In the final scenario, using only absolute cases, those with known positive and negative measles IgG titre (*n* = 1447), gave us a *p,* seropositive rate of 93%, and the calculated p_c_ based on Plans’ indicator of 89.1%. Herd immunity for the 1447 absolute cases was established since *p* > p_c_. Subsequent analysis by age, gender and ethnic subgroups revealed similar findings as in the best case scenario when *p* was also 93%.

## Discussion

The positive seroprevalence of measles IgG antibodies in Seremban was 87%. This is higher compared to a previous Malaysian study by Saraswathy et al. [10] where individuals aged more than 7 years had prevalence rate of 82.8%. A study conducted among healthy individuals in Baoji City, Shaanxi Province, China demonstrated the overall measles antibodies positive rate of 78.9% [14]. However, the seropositive rate was lower when compared to a study conducted in Catalonia, Spain where the global measles antibodies was 98.3% [7]. Contrary to this study, the study in Catalonia, Spain was conducted as a population based study which represents the whole community. In our study, the respondents were limited to those who attended government or public health clinics.

Factors that were explored among all respondents in this study included socio-demographic characteristics such as age, gender, ethnicity, education level, household income, place of birth, and location of place of birth and health clinics attended. Factors that may contribute to failure of being immune or getting immunization such as history of medical illness and medication history were also investigated. In the multivariable analysis, after adjusting for all explored confounders, we found only age group remained the significant predictors for measles antibodies in this study.

The seroprevalence of measles antibodies found in children (90%) was slightly lower than adults group (94%) but much higher than in the adolescents and young adults group (74%). Comparing to children aged 6–9 years in this study, adolescents and young adults group had OR of 0.31 and adults group had increased OR of 1.74 to have positive measles antibodies. Similar findings were elicited in a study conducted in Catalonia, Spain [8] and Israel [15]. However, the prevalence rate for measles antibodies in those studies among adolescents and young adults were higher (83.8% and 85.7% respectively) than that found in this study. The difference in these findings may be due to the supplementary immunization activities (SIA) that were carried out in Catalonia in 1998 and 1999 for the cohorts born in 1988–1999.

Furthermore, the low prevalence rate of positive measles antibodies and high prevalence rate of indeterminate in teenagers and young adults aged 15–24 years in this study may be due to primary or secondary vaccine failure.

Primary vaccine failure or failure to seroconvert could happened among adolescents and young adults in Malaysia as their birth cohort received immunization at a very young age which was at 9 months old. Leuridan et al. [16] explained that vaccination at an earlier age is not advisable because of immature human responses of infants.

Secondary vaccine failure or waning immunity could be another reason for the decline of measles antibodies in both children and adolescents and young adults groups as compared to adults group. Vaccine recipients would be most likely to develop this type of vaccine failure because those who are vaccinated have lower measles-specific antibody as compared to those who experienced natural measles infection. In addition, the decline in measles antibodies is more rapid in those who are vaccinated than those who recuperate from measles infection [17]. The finding of this current study did not differ from a prospective cohort study by LeBaron et al. which revealed a progressive decline in measles antibodies 10 years after a second dose of measles vaccine [18]. This could explained why adolescents and young adults aged 15–24 years in this study had lower prevalence rate of measles antibodies than children 6–9 years old.

The current study showed there were no significant differences between genders in term of measles antibodies level. This result was in line with seroprevalence surveys conducted by Levine et al. [15], Sultana et al. [19], Yekta et al. [20], Davidkin et al. [21] and Onoja & Adeniji [22] but differ from a study by Poethko-Muller & Mankertz [23] which found boys had higher levels of susceptibility than girls. Aaby et al. [24] also found that there were sex differences in measles vaccine efficacy in their study.

Several epidemiological studies have identified ethnicity as one of the factors that prevented certain groups of people from being vaccinated and resulted in low prevalence of measles antibodies [25–27]. A study by Mixer et al. [28] revealed different ethnicity with different cultural background is an important predictor for the uptake of MMR vaccine. White mothers staying in an underprivileged area were not keen to get their child immunized [29]. McQuillan et al. [30] demonstrated that non-Hispanic whites and Mexican Americans were the most susceptible group for measles infection in the United States population. Contrary to those findings, this study did not show any significant association between ethnicity and seropositive measles antibodies.

Respondents with lower education were found to have higher seropositive rate than those with higher education from the initial crude analysis. However, multivariate analysis revealed there was no association between level of education and positive measles antibodies. A seroprevalence study by Levine et al. [15] also showed similar findings but McQuillan et al. [30] found that higher education was a predictor of seropositive measles antibodies.

In the current study, total household income among respondents either lower or higher had no significant association with seropositive measles antibodies. The finding was similar to the study conducted by Sultana et al. [19] where the association between prevalence of measles antibodies and socio-economic classes was not significant. In contrast with this finding, in the Catalonian study, seropositive measles antibodies were higher among the rich compared to the poor [7]. Another study by Mixer et al. [28] also showed that higher MMR vaccination coverage was significantly associated with higher socioeconomic status.

McQuillan et al. [30] concluded in their study that place of birth outside of the United States was a predictor for positive measles antibodies. United States-born Mexican Americans had higher seropositivity for measles antibodies than foreign-born Mexican Americans. Senn et al. [31] demonstrated in the study conducted in Papua New Guinea that urban population were more susceptible to get measles infection as compared to rural population. Foreign-born children in Germany also had a higher risk of susceptibility against measles [32]. However, the present study did not find any association between positive measles antibodies titre and place of birth (either in or outside Seremban) or rural or urban locality of birth. The location of health clinics attended was also not a significant factor for development of measles antibodies in this study. Onoja & Adeniji [22] also found non-significant difference between the mean measles titre in two locations of health centres in Nigeria.

There were no demonstrated differences between juvenile idiopathic arthritis patients and healthy individuals in term of measles antibodies concentration and seroprotective rates [33]. The finding was similar with the present study in which medical problems and medication history were not significant predictors for seropositive measles antibodies. Nevertheless, seropositivity can be expected in the majority of vaccine recipients with DiGeorge Syndrome, post-bone marrow or solid organ transplantation and in HIV-positive children with no severe immune defects [34]. High levels of measles antibodies were also documented in psychiatric patients on treatment compared to non-psychiatric individuals [35]. Prospective cohort study should be considered in investigating contributing factors for elevated measles antibodies in such groups.

### Establishment of herd immunity against measles among all respondents

Plans [8] developed a new indicator to assist in determining the accepted level of herd immunity in a population. The new indicator which is the critical prevalence of antibodies associated with herd immunity (p_c_) has enabled a population to be evaluated for the establishment of herd immunity. This indicator was derived from the prevalence of positive measles antibodies (*p*) obtained in a seroprevalence study. Taken into consideration is the value of basic reproductive number, R_o_ which varies according to population density. A higher value of R_o_ and p_c_ is expected in a dense population [8].

In this study, herd immunity was not established in analysis of the total study population (*n* = 1541). Herd immunity against measles was established in children, adults, Chinese and others (other than Malay, Indian and Chinese ethnicity) but not in adolescents and young adults, both genders, and among Malays and Indians. Based on multivariable analysis, age group was the only predictor for positive measles antibodies in this study. Thus, it is worth planning an immunization program focusing on the susceptible age group which is the adolescents and young adults. Future outbreaks are forecasted imagining these groups of individuals reaching adulthood. Similar findings were reported in many of the previous measles outbreaks in Malaysia [36]. The adolescents and young adults group can be considered a homogenous population because many remain in secondary schools, colleges and universities. Supplementary immunization activities (SIA) can be concentrated among secondary school students and freshmen in universities.

The three different age groups in this study came from various birth cohorts experiencing different immunization programs or strategies implemented in the country as shown in Table 3. From the table, we hypothesize that adults aged 45–54 years developed their immunity secondary to natural infection. Vaccination was lacking and wide circulation of measles virus in the 1960s had ended in a near-universal measles exposure and enhanced the establishment of natural immunity. On the other hand, children, adolescents and young adults groups developed their immunity most probably via vaccination. The low prevalence rate of positive measles antibodies and high prevalence rate of indeterminate in teenagers and young adults aged 15–24 years may be attributable to different vaccination strategies during childhood.

**Table 3:** Malaysian immunization program by birth cohort

Age group (years)	Birth cohort	Immunization program in Malaysia
45–54	Born in 1960–1969	• No measles vaccination program in Malaysia
15–24	Born in 1990–1999	• Single dose measles vaccination was given to children at 9 months old • Malaysia mass immunization campaign or catch-up campaign in 2004 in which another booster dose of single measles vaccine was given to all school children aged 7–15 years old (Birth cohort 1990–1997 in this study were involved) • Children who were born in 1998–1999 received second dose of MMR vaccine when they were in standard one
6–9	Born in 2006–2009	• Received two doses MMR vaccine; once at 12 months and the second at 7 years of age

The three scenarios with different assumptions made for indeterminate serological status in Table 2 can be eye opening for health managers and policy makers to strengthen the measles control strategies in Malaysia. Taking into account the best case scenario, where all indeterminate cases were considered seropositive, the establishment of herd immunity against measles is seen in the whole population, in children and adults group, in both gender and in all ethnic groups but not among adolescents and young adults. It has been a decade since the last catch-up campaign for measles which was in 2004. Again, catch-up campaigns need to be implemented in Malaysia in order to block the transmission of the measles virus.

With this finding, policy makers and health managers can plan new immunization strategies for Seremban district that should include catch-up campaigns. The result of this study may be generalized to populations with similar background as ours. Studies on the cost effectiveness of different immunization strategies such as vaccination of adults, catch-up vaccination and different timing of vaccination should be conducted to help in evidence-based decision making. Several study limitations were identified and these include selection and measurement or information bias. The study is limited to clients who attended public or government health clinics. A population based study similar to the samples in the Malaysian National Health Morbidity Surveys (NHMS) would be more representative and generalizable. Response bias should be considered as those who agreed to participate in this study may in some ways be different from those who refused to participate. However, this type of bias had been minimised due to our high response rate.

## Conclusions

In order to achieve control over measles, it is crucial to implement evidence-based vaccination policy. This study recognized adolescents and young adults as pocket of susceptibles. In future, supplementary immunization activities (SIAs) in Malaysia should focus on adolescents and young adults. Seroprevalence study should be carried out at regular interval to monitor measles antibodies in different birth cohorts in order to identify to whom intervention strategies should be prioritized. This will eventually provide better measles control and ultimately measles elimination in Malaysia.

## Abbreviations

CI: Confidence interval
ELISA: Enzyme-linked immunosorbent assay
HIV: Human Immunodeficiency Virus
IgG: Immunoglobulin G
MCV: Measles containing vaccine
MMR: Measles-mumps-rubella
NHMS: National Health Morbidity Surveys
OR: Odds ratio
PPV: Positive predictive value of serological test
SIAs: Supplementary immunization activities
SPSS: Statistical Package for Service Solutions
UKMMC: Universiti Kebangsaan Malaysia Medical Centre

## References

[1] Strebel PM, Cochi SL, Hoekstra E, Rota PA, Featherstone D, Bellini WJ, Katz SL (2011). A world without measles. J Infect Dis.

[2] CDC (2008). Progress in Global Measles Control and mortality reduction, 2000-2007. Morb Mortal Wkly Rep.

[3] CDC (2013). Progress toward Measles Elimination-Western Pacific Region, 2009-2012. Morb Mortal Wkly Rep.

[4] MOH (2006). Measles Prevention and Control in Malaysia: Handbook for healthcare personnel.

[5] Hollingsworth TD (2009). Controlling infectious disease outbreaks: Lessons from mathematical modelling. J Public Health Policy.

[6] De Serres G, Gay NJ, Farrington CP (2000). Epidemiology of transmissible diseases after elimination. Am J Epidemiol.

[7] Dominguez A, Plans P, Costa J, Torner N, Cardenosa N, Batalla J (2006). Seroprevalence of measles, rubella, and mumps antibodies in Catalonia, Spain: results of a cross-sectional study. Eur J Clin Microbiol Infect Dis.

[8] Plans P (2010). Prevalence of antibodies associated with herd immunity: A new indicator to evaluate the establishment of herd immunity and to decide immunization strategies. Med Decis Mak.

[9] Pocock SJ (1983). Clinical Trials: A Practical Approach.

[10] Saraswathy TS, Zahrin HN, Norshahmimi H, Az-Ulhusna A, Zainah S, Rohani J (2009). Impact of measles elimination strategy on measles incidence in Malaysia. Southern Asian J Trop Med Public Health.

[11] Novatech Immunodiagnostica GMSH. http://www.novatec-id.com/. Accessed 20 August 2013.

[12] Anderson RM (1992). The concept of herd immunity and the design of community-based immunization programmes. Vaccine.

[13] Georgette N (2007). The quantification of the effects of changes in population parameters on the herd immunity threshold. Int J Epidemiol.

[14] Zhang X, Kou G, Du H, Ju Z, Zhong L, Cui X (2013). Measles epidemiology and survey of measles immunity level among healthy population in Baoji City, Shaanxi Province, China. Jpn J Infect Dis.

[15] Levine H, Zarka S, Ankol OE, Rozhavski V, Davidovitch N, Aboudy Y (2015). Seroprevalence of measles, mumps and rubella among young adults, after 20 years of universal two-dose MMR vaccination in Israel. Human Vaccines Immunother.

[16] Leuridan E, Sabbe M, Damme PPV (2012). Measles outbreak in Europe: Susceptibility of infants too young to be immunized. Vaccine.

[17] Markowitz LE, Preblud SR, Fine PE, Orenstein WA (1990). Duration of live measles vaccines-induced immunity. Pediatr Infect Dis J.

[18] LeBaron CW, Beeler J, Sullivan BJ, Forghani B, Bi D, Beck C, Audet S, Gargiullo P (2007). Persistence of measles antibodies after 2 doses of measles vaccine in a postelimination environment. Arch Pediatr Adolesc Med.

[19] Sultana R, Rahman MM, Hassan Z, Hassan MS (2006). Prevalence of IgG antibody against measles, mumps and rubella in Bangladeshi children: A pilot study to evaluate the need for integrated vaccination strategy. Scand J Immunol.

[20] Yekta Z, Porali R, Taravati MR, Salary S, Khalily F, Shahabi S (2007). Measles IgG sero-prevalence and its attributable factors in 5-25-year-old cases prior mass vaccination campaign in Urmia, Northeastern Iran. Iranian Red Crescent Med J.

[21] Davidkin I, Jokinen S, Broman M, Leinikki P, Peltola H (2008). Persistence of measles, mumps and rubella antibodies in an MMR-vaccinated cohort: a 20-year follow-up. J Infect Dis.

[22] Onoja AB, Adeniji AJ (2013). Kinetics of measles antibody by hemagglutination inhibition assay in children in south-west and north-central Nigeria. Int J Infect Dis.

[23] Poethko-Muller C, Mankertz A (2011). Sero-epidemiology of measles specific IgG antibodies and predictive factors for low or missing titres in a German population-based cross sectional study in children and adolescents (KiGGS). Vaccine.

[24] Aaby P, Martins C, Bale C, Garly ML, Rodrigues A, Biai S, Lisse IM, Whittle H, Benn CS (2010). Sex differences in the effect of vaccines on the risk of hospitalization due to measles in Guinea-Bissau. Paediatr Infect Dis J.

[25] Stein-Zamir C, Abramson N, Shoob H, Zentner G (2008). An outbreak of measles in an ultra-orthodox Jewish community in Jerusalem, Israel, 2007- an in-depth report. Eurosurveillance.

[26] Lernout T, Kissling E, Hutse V, De Schrijver K, Top G (2009). An outbreak of measles in orthodox Jewish communities in Antwerp, Belgium, 2007–2008: different reasons for accumulation of susceptibles. Eurosurveillance.

[27] Anis E, Grotto I, Moerman L (2009). Measles in a highly vaccinated society: the 2007–08 outbreaks in Israel. J Infect.

[28] Mixer RE, Jamrozik K, Newsom D (2007). Ethnicity as a correlate of the uptake of the first dose of mumps, measles and rubella vaccine. J Epidemiol Community Health.

[29] Baker D, Garrow A, Shiels C (2011). Inequalities in immunisation and breast feeding in an ethnically diverse urban area: cross-sectional study in Manchester, UK. J Epidemiol Community Health.

[30] McQuillan GM, Kruszon-Moran D, Hyde TB, Forghani B, Bellini W, Dayan GH (2007). Seroprevalence of measles antibody in the US population, 1999–2004. J Infect Dis.

[31] Senn N, Riddell M, Omena M, Siba P, Reeder JC, Clements CJ, Morgan C (2009). Measles in Papua New Guinea: an age-specific serological survey. Vaccine.

[32] Poethko-Muller C, Mankertz A (2012). Seroprevalence of measles-, mumps- and rubella-specific igg antibodies in German children and adolescents and predictors for seronegativity. PLoS ONE.

[33] Heijstek MW, Gageldonk PGM, Berbers GAM, Wulffraat NM (2012). Differences in persistence of measles, mumps, rubella, diphtheria and tetanus antibodies between children with rheumatic disease and healthy controls: a retrospective cross-sectional study. Ann Rheum Dis.

[34] Gluck T, Muller-Ladner U (2008). Vaccination in patients with chronic rheumatic or autoimmune diseases. Clin Infect Dis.

[35] Dickerson F, Stallings C, Origoni A, Copp C, Khushalani S, Yolken R (2010). Antibodies to measles in individuals with recent onset psychosis. Schizophr Res.

[36] Rosemawati A (2003). Towards measles elimination – measles situation in Malaysia before. Infect Dis Bull.

